# Density-dependent growth in ‘catch-and-wait’ fisheries has implications for fisheries management and Marine Protected Areas

**DOI:** 10.1007/s13280-019-01158-1

**Published:** 2019-03-09

**Authors:** Julian Merder, Patricia Browne, Jan A. Freund, Liam Fullbrook, Conor Graham, Mark P. Johnson, Alina Wieczorek, Anne Marie Power

**Affiliations:** 1 Institute for Chemistry and Biology of the Marine Environment, University of Oldenburg, Oldenburg, Lower Saxony Germany; 2 Ryan Institute, National University of Ireland Galway, University Road, Galway, Ireland; 3 Marine & Freshwater Research Centre, Galway-Mayo Institute of Technology, Dublin Road, Galway, Ireland

**Keywords:** Body size, Competition, Density compensation, Marine Protected Areas, Resource limitation, Small-scale fisheries

## Abstract

Stock enhancement activities provide an opportunity to examine density-dependent suppression of population biomass which is a fundamental issue for resource management and design of no-take-zones. We document ‘catch-and-wait’ fisheries enhancement where all but the largest lobsters are thrown back, recapturing them later after they have grown to a larger size. The residency, rate of return, and potential negative density-dependent effects of this activity are described using a combination of tagging and v-notching and by relating spatial growth patterns to population density defined with Catch Per Unit Effort. The results successfully demonstrated the concept of catch-and-wait practices. However, a density-dependent suppression of growth (in body size) was observed in male lobsters. This demonstrates a mechanism to explain differences in lobster sizes previously observed across EU fishing grounds with different stock densities. This negative effect of density could also affect individual biomass production in marine reserve or no-take zones.

**Electronic supplementary material:**

The online version of this article (10.1007/s13280-019-01158-1) contains supplementary material, which is available to authorized users.

## Introduction

Fisheries can be considered as ecological experiments, potentially informative about population dynamics and ecosystem structure, and inferences from fisheries about density dependence in population regulation can be made in various ways (Chen et al. 2005; reviewed in Jensen et al. 2012). While density dependence clearly impacts the estimation of potential fishery yields, it can also potentially limit the benefits of marine reserves and no-take zones because protections can increase densities inside reserves and lead to reduced body growth as a consequence of density dependence (Gårdmark et al. 2006). However, empirical evidence for this appears limited and there are many studies demonstrating fisheries benefits from marine reserves (Gell and Roberts 2003; Halpern 2003; Gaines et al. 2010). We present an assessment of an undescribed fisheries enhancement activity associated with small-scale creel (pot) fisheries in inshore areas of Ireland’s coast, including associated density-dependent effects. This practice, which we term ‘catch-and-wait’, describes capturing *Nephrops norvegicus* (Linnaeus 1758) lobsters and throwing back all but the largest individuals, including those which are above legal landing size of 25 mm carapace length (CL), only to capture these at a later point when they have achieved larger size. This is a form of stock enhancement as returned individuals add to the spawning stock biomass when individuals are left on the grounds for long enough to reproduce (Bell et al. 2008).

Catch-and-wait is distinct from ‘ranching’ because the latter refers to releasing cultured individuals into the sea for on-growing (Bell et al. 2008). At present, closure of the aquaculture lifecycle in *Nephrops* is too inefficient to consider ranching (Powell and Eriksson 2013). Even when supply from aquaculture is feasible, proof-of-concept for ranching is recommended, e.g. tagging stocked individuals to track survival into the adult spawning stock and justify the cost of production (Munro and Bell 1997). Importantly, releases should also be carried out in a way that does not cause density-dependent mortality among the released animals or replacement of wild juveniles (Bell et al. 2008).

Reports from fishermen suggest that catch-and-wait ‘works’, but the residency of individuals and the rate of return have not yet been documented. The potential for negative density-dependent effects as stock sizes increase is unknown, but there are reasons to suspect such effects in this lobster’s populations. *Nephrops* is a mud-dweller, sheltering within burrows (Lauria et al. 2015; Sbragaglia et al. 2017) and mud habitats may be a limiting resource for this species. A dome-shaped response has been demonstrated between burrow density and habitat quality (expressed as the percentage silt plus clay of the sediment). A proposed mechanism for this relationship is that increasingly elaborate burrows begin to interfere with each other above a threshold habitat quality, such that densities drop in the most optimal substrates for burrowing (Campbell et al. 2009). Density dependence may also be evident in *Nephrops* stock-specific growth curves (Tuck et al. 1997). Theory predicts that resource limitation affects asymptotic body length (*L*_max_) rather than growth rate, ‘*k*’, in the von Bertalanffy growth function (Beverton and Holt 1957; Gårdmark et al. 2006). Indeed, Johnson et al. (2013) showed that theoretical maximum body size, *L*_max_, was negatively related to population density in fishing grounds across Europe.

Direct evidence of density dependence in marine populations is rare (Wahle 2003), and some authors argue that it is unclear whether adults of exploited marine populations are resource limited (Sanchez-Lizaso et al. 2000). Datasets estimating density limitations of growth rates in commercially exploited marine species are therefore particularly valuable. We also have no information on how individual lobsters behave on fishing grounds during enhancement activities. In the present study, we demonstrated the potential for catch-and-wait fisheries in *Nephrops*, showing high retention at the release site and no differences in distance travelled from the point of release between sexes, starting sizes, or amount of time spent at liberty. Distance travelled from the release point also had no effect on growth (expressed as increase in body size) for either sex. However, a density-dependent reduction was demonstrated in the growth of males in high density patches. Reduced growth (hence body size) at high densities could therefore explain the differences in maximum/mean body sizes of *Nephrops* across European grounds.

## Materials and methods

### Study site and fishing

**Fig. 1:**
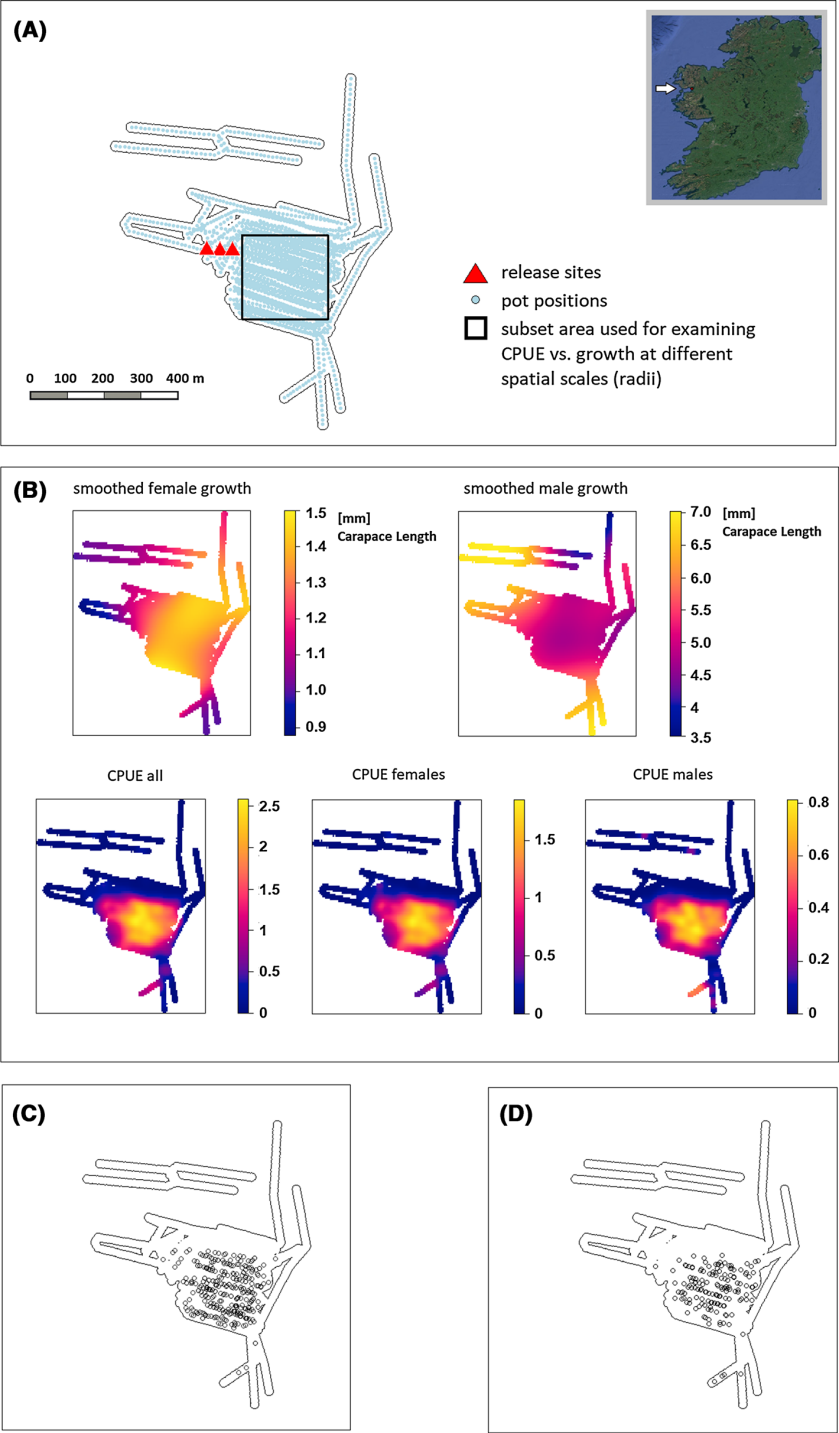
**a** Clew Bay study area with creel positions and release sites of tagged *Nephrops norvegicus*. Mapping was done with QGIS (QGIS Development Team 2017) and the “spatstat” package in R (Baddeley et al. 2015; R Core Team 2017). **b** Kernel density estimation of total CPUE and kernel density-based smoothed growth for tagged males and females (Bandwidth 70 m). Note that because growth rates are smoothed, and there were no recaptures at the extremities of the fished area (in 1**b**), the pattern reflects the lower growth rates at the edge of the main patch **c** locations of v-notched recaptured females, **d** locations of v-notched recaptured males

The catch-and-wait fishery is creel-based and located in the inshore grounds of Clew Bay in Ireland. Clew Bay covers an area of approximately 31 259 ha and is close to, but outside, the Aran Grounds *Nephrops* Functional Unit (of assessment). A total of 1177 *Nephrops* individuals were captured, tagged, and released at the study site over the course of the experiment. To overcome the problem of moulting, internal tags were used (sequential Coded Wire Tags, Northwest Marine Technology Inc.), which have shown no negative effects in this species (Fullbrook et al. 2017). The tagged individuals were released at three sites approximately 30 m apart on three successive dates in 2013 (5, 17, and 19 June) (Fig. 1a). These individuals were dropped to the seabed (18-20 m deep) in a controlled manner (see Haynes et al. 2016) and allowed to escape at will over 72 h. The fishing grounds were left fallow (no fishing) until April to September 2014, when fishing to recapture individuals was carried out by creel fishing. On every recapture day, up to 6 ‘strings’ equipped with 48 creels (pots) separated regularly every 10 m were baited with salted herring, dropped to the seafloor, and left to soak for 48 h (occasionally 72 h) before being hauled back in. Additional recapture fishing was carried out approximately 2 years post-release in June and July 2015. All *Nephrops* including non-tagged individuals in every pot were counted in every fishing trip to provide a spatial estimate of population ‘density’ defined as Catch Per Unit Effort (CPUE, mean individuals pot^−1^). However, once counted and measured, all individuals were returned apart from tagged recaptures which were returned to the laboratory for dissection of tags. For the tagged individuals, the following parameters were recorded: starting size (mm carapace length ‘CL’), growth, i.e. increase in CL between tagging and recapture (mm CL) and the distance travelled at recapture (m) from the release point. Further details of the tag and release experiment are described in Haynes et al. (2016). Raw data, including detail about sizes of all individuals, tagged and untagged, with growth measurements of tagged individuals, are given in Power et al. (in press).

In the same area as above, v-notching was carried out during April and May 2014 on 1309 individuals (*n* = 473 male and *n* = 836 female). All individuals were notched over a range of sizes from 15 to 55 mm CL (female) and 25 to 65 mm CL (male). The outer uropod of the telson was marked with a small notch on the bottom edge using a ‘lobster v-notcher plier’ (GT Products Marine Store, United Kingdom). Fishing to recapture notched individuals took place from May to July 2014.

### Data analysis

#### Spatial distribution of recaptures and distance travelled

Released individuals are potentially disorientated and may be at greater risk of mortality as they build or search for a burrow and interact with resident animals. Individuals may be forced to peripheral habitats, even leaving the fished mud patch, if residents defend territories. A null model would be that released individuals disperse over the fishing ground finding or building burrows in proportion to the existing population and suffering no greater mortality. Under this null model, the number of tagged individuals recovered should increase in linear proportion to the total catch, with deviations from linearity indicating clustering, while declines in the proportion of tagged *Nephrops* in the catch would indicate higher mortality, or migration away from the area, for released animals. Deviations from the null model were evaluated using plots of cumulative numbers of tagged recaptures against total catch of *Nephrops* over the whole survey. We calculated the root mean squared error (RMSE) from this null model as the measure of spread between the observed values and a line representing a constant recapture rate with zero intercept and a slope estimated from the ratio of total catch: total tagged recaptures. Observed RMSE was compared to values obtained by permutation (1000 repetitions) of tagged recaptures across all capture locations. If the observed value lies within the 95% confidence limits of the permutations, this supports the null hypothesis about the distribution of tagged individuals with no losses due to higher mortality or individuals leaving the study area.

The distances moved by released individuals are likely to reflect the search strategy for obtaining a new burrow and interactions with residents. The distance moved between release and capture may be influenced by sex (e.g. generally more fighting occurs between males) or size (e.g. due to trade-offs between mortality risk and search time or due to changes in the probability of winning burrow fights). Number of years of liberty may also be a factor if released individuals remain transient on the grounds for long periods. Distances between release point and recapture were therefore split by time at liberty: 1 or 2 years, then by sex: male, female and finally by starting size: big, small (defined as > or < sex-specific median of starting size); which resulted in a total of eight different groups. The presence of any differences in median distance moved between groups was assessed using a Kruskal–Wallis (KW) test. The KW test was repeated across the same groups using high and low growth (mm CL) instead of starting size in case higher growth was associated with finding a burrow quickly (and presumably with less crossing of the fishing ground) after release.

#### Density-dependent effects on growth and survival

An expectation for the distribution of *Nephrops* across a mud patch is that densities are higher in more suitable habitat. The more suitable habitat could result in higher growth and survival. However, higher densities could also negatively impact some or all individuals through competitive interactions. A final alternative is that densities may have no net effect on growth and survival as individuals move to maximise these aspects of fitness by a trade-off between habitat quality and density-dependent competition [i.e. conforming to an ideal free distribution Fretwell and Lucas (1970)]. To summarise the spatial distribution of all males and females (CPUE), we applied kernel density estimations using the “spatstat” package in R (Baddeley et al. 2015; R Core Team 2017). Details can be found in Supplementary Material. The spatial pattern of growth in tagged recaptures was visualised using a spatial smoothing based on the Nadaraya–Watson smoother (Watson 1964; Nadaraya 1989) implemented in the “spatstat” package (Baddeley et al. 2015).

Competitive interactions between *Nephrops* individuals are likely to be related to foraging excursions from the burrow, but these movements are not well characterised. We therefore needed to analyse growth at a range of spatial scales, adopting the approach from Gunton and Kunin (2009) previously used to analyse the influence of density on survival and reproduction in plants and combined with common point pattern methods (Wiegand and Moloney 2014) and analysis of spatial stratified heterogeneity (Wang et al. 2016). The small number of individuals with negative growth were excluded from this analysis, and the analysis was restricted to an area with more homogenous levels of sampling effort (black rectangle in Fig. 1a).

**Fig. 2:**
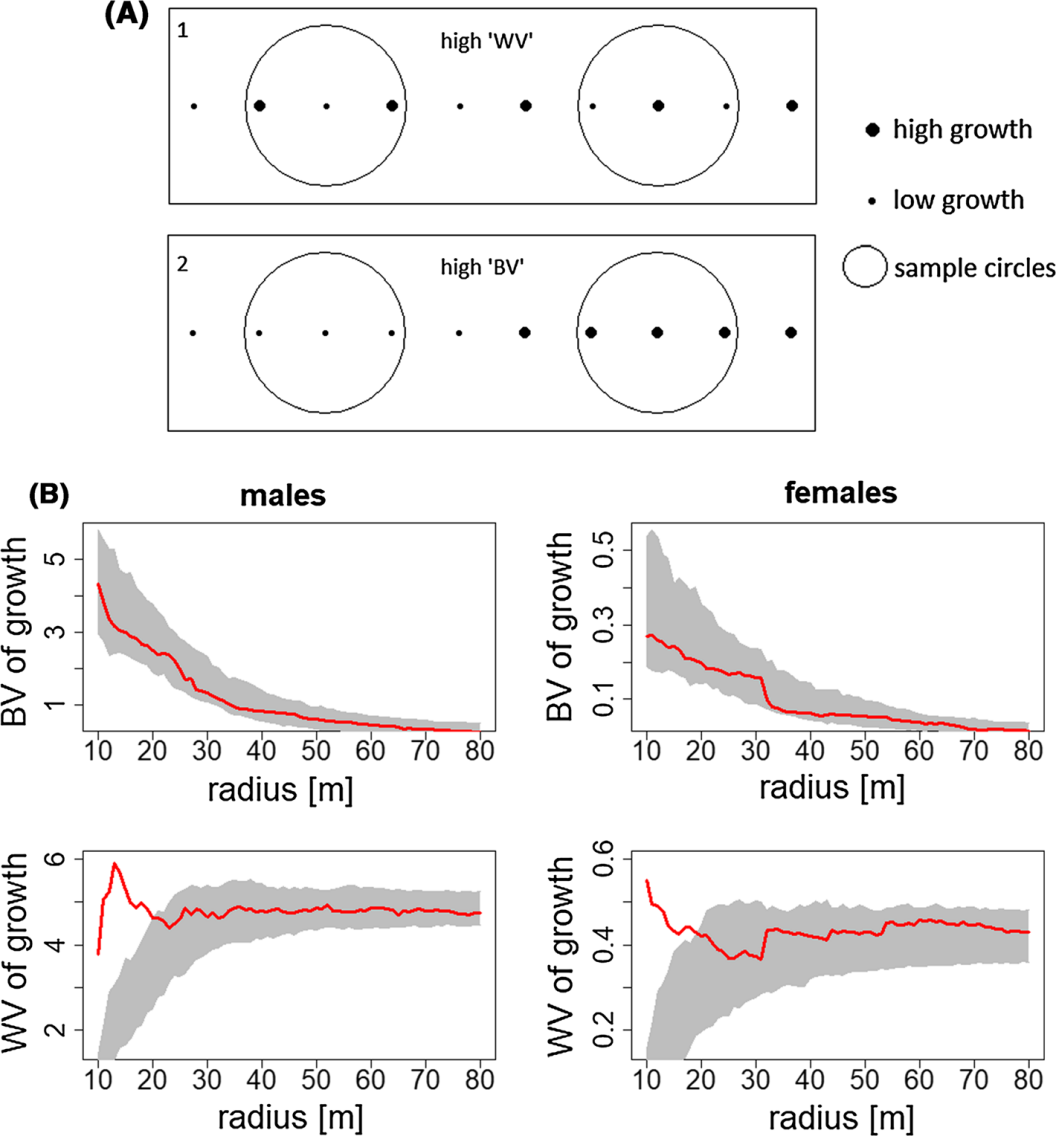
**a** Example of spatial pattern resulting in high within-circles variance in growth ‘WV’ (1) or high between-circle variance ‘BV’ in growth (2). **b** Left: ‘BV’ and ‘WV’ along different radii for males. Right: For females. Greyed area shows 2.5–97.5% percentile of BV (or WV) distribution calculated with permutation tests, red lines indicate sample values

Our scale-dependent approach was based on sampling circles of varying radii centred on the spatial positions of every recaptured individual bearing a tag. Circles were inflated by increasing radii in 1 m increments, ranging from a minimum of 10 m, which roughly corresponds to the fishing radius of a single pot, up to a maximum radius of 80 m. The effects of spatial scale on variability in mean growth of tagged individuals were evaluated using the mean within-circle variance (across all circles, ‘WV’) and the between-circles variance (‘BV’), for every radius as follows:

*WV* This defined the variance inside every circle at a given radius and computed the mean of these variances. A high WV indicates that growth shows a high variability at a particular scale and that this variability is high over all circles. A high WV could reveal a spatial pattern like the one shown in Fig. 2a (1) with high and low growth individuals found together.

*BV* This defined mean growth in every circle at a given radius and computed the variance between these mean growth values. A high BV indicates that circles show a high difference in mean growth, which might be attributable to a spatially heterogeneous growth pattern, as illustrated in Fig. 2a (2).

The statistical significance of BV and WV values across the range of radii were determined by reference to values obtained by permutative resampling (1000 times). Permutations shuffled growth values while retaining the relation between pots and number of individuals. This ensured CPUE was held constant inside sampling circles and only growth values changed randomly in each iteration. WV and BV curves (for varying radii along the abscissa) based on the observed data were plotted, along with grey shaded bands indicating the 2.5–97.5% percentile range of the permuted values. A WV value above the 97.5% percentile reflects an accumulation of highly dissimilar growth increments in individuals inside a sampling circle, while a value below the 2.5% percentile expresses a higher aggregation of homogeneously growing individuals in a sampling circle than would be expected due to chance. As sampling circles were set up on every individual, we checked if the degrees of freedom in variance calculations influenced the results, by repeating the analysis using between sum of squares and within sum of squares instead of the variances.

Density-dependent effects on growth were examined using the correlation between mean growth and CPUE. This analysis was also carried out across a range of scales, with CPUE inside a sampling circle based on the complete catch of *Nephrops* divided by the number of pots inside the circle. This was initially carried out separately for males and females before repeating the analysis using total CPUE values. As growth in male *Nephrops* is higher in smaller male individuals (smaller starting sizes, Haynes et al. 2016), we conducted an additional analysis using a semipartial correlation controlling for starting size. After reviewing our results, we repeated our analysis using the upper and lower quartile of growth in a sampling circle along all radii instead of the mean. Similar to the BV and WV analyses, permutation tests (1000 iterations) were used to examine the departure of observed correlation coefficients (CPUE vs. growth) from spatially random pattern.

Survival of *Nephrops* can be inferred from the recovery of v-notched individuals. Assuming that the density is a static pattern with constant density-independent mortality across the grounds, immigrants (v-notched individuals returning to the seabed) will be found in proportion to the size of the local population. The correlation between v-notched recaptures and non-v-notched captures per pot was tested using the modified *t* test to account for spatial autocorrelation (Clifford et al. 1989; Dutilleul 1993) implemented in the “SpatialPack” package of “R” (Vallejos et al. 2018).

## Results

### Spatial distribution of recaptures and distance travelled

In 2014, a total of 204 tagged individuals (98 males and 106 females) were recaptured. Besides this, untagged males (*n* = 1457) and females (*n* = 3328) were captured for CPUE calculation. Cumulative recapture rate was proportional to the total capture rate with RMSE from observations not significantly different from randomly permuted RMSE values (Suppl. Fig. S1). This implies that tagged individuals did not return to any particular part of the ground and there were no spatial difference in losses due to mortality/emigration from the study area. An additional 36 individuals were recovered over ten sampling days in 2015, indicating that survival/residency persisted over time.

Recaptured individuals had moved between 21 and 536 m from the point of release. The distance travelled between release and capture points did not seem to vary by sex, year, or size class (Kruskal–Wallis test, *H* = 6.52, df = 7, *p* = 0.480; Table 1). Repeating the test using high or low growth instead of starting size did not change the outcome (*H* = 10.65, df = 7, *p* = 0.154).

**Table 1:** Distance travelled by tagged *Nephrops norvegicus* from release site. Categories “Big” and “Small” are based on upper and lower median starting size distribution

	Distance (m) travelled after 1 year (*n* = 204)			Distance (m) travelled after 2 years (*n* = 36)		
	Median	Range	SD	Median	Range	SD
Males						
Big	213.2	60.4–394.4	81.8	234.2	181.2–450.6	106.3
Small	216.3	63.7–360.2	84.6	308.4	223.5–362.7	58.4
Females						
Big	223.4	41.7–536.2	97.5	182.4	60.8–514.9	131.5
Small	215.9	21.3–375.7	84.0	224.5	121.6–403.7	83.3

### Density-dependent effects on growth and survival

Catches were highest in the centre of the fished area, indicating a fairly discrete patch of suitable mud habitat. Kernel density estimations of CPUE did not suggest any difference in the distribution of males and females (Fig. 1b). In contrast, the spatial distributions of smoothed growth did appear to differ between sexes. Male growth was higher in peripheral areas compared to the central area where catches were higher. Females had the opposite pattern, with higher growth tending to be in the central fished area (Fig. 1b).

The variance of mean growth rate in sampled circles (BV) declined as the radius of circles increased, decreasing to approximately zero (Fig. 2b). This decrease would be expected from sampling a random distribution. The confidence limits from permutations suggested that neither sex had any spatial structure in growth rates across the centre of the fished ground. Note that the BV test was restricted to the most sampled area to avoid edge effects and therefore is not able to pick up the apparent differences between centre and periphery in Fig. 1b.

Local variances in growth rate (WV) were higher in small sample circles than would be expected by chance (Fig. 2b, lower two graphs). This suggests possible competition at small scales with similar-sized individuals avoiding each other or dominance by larger individuals suppressing growth in smaller *Nephrops*. WV in females declined with circle radius, while WV in males initially increased to a maximum at around 15 m before falling again. Repeating the analysis using between sum of squares and within sum of squares instead of variances (BV & WV) did not affect these conclusions.

**Fig. 3:**
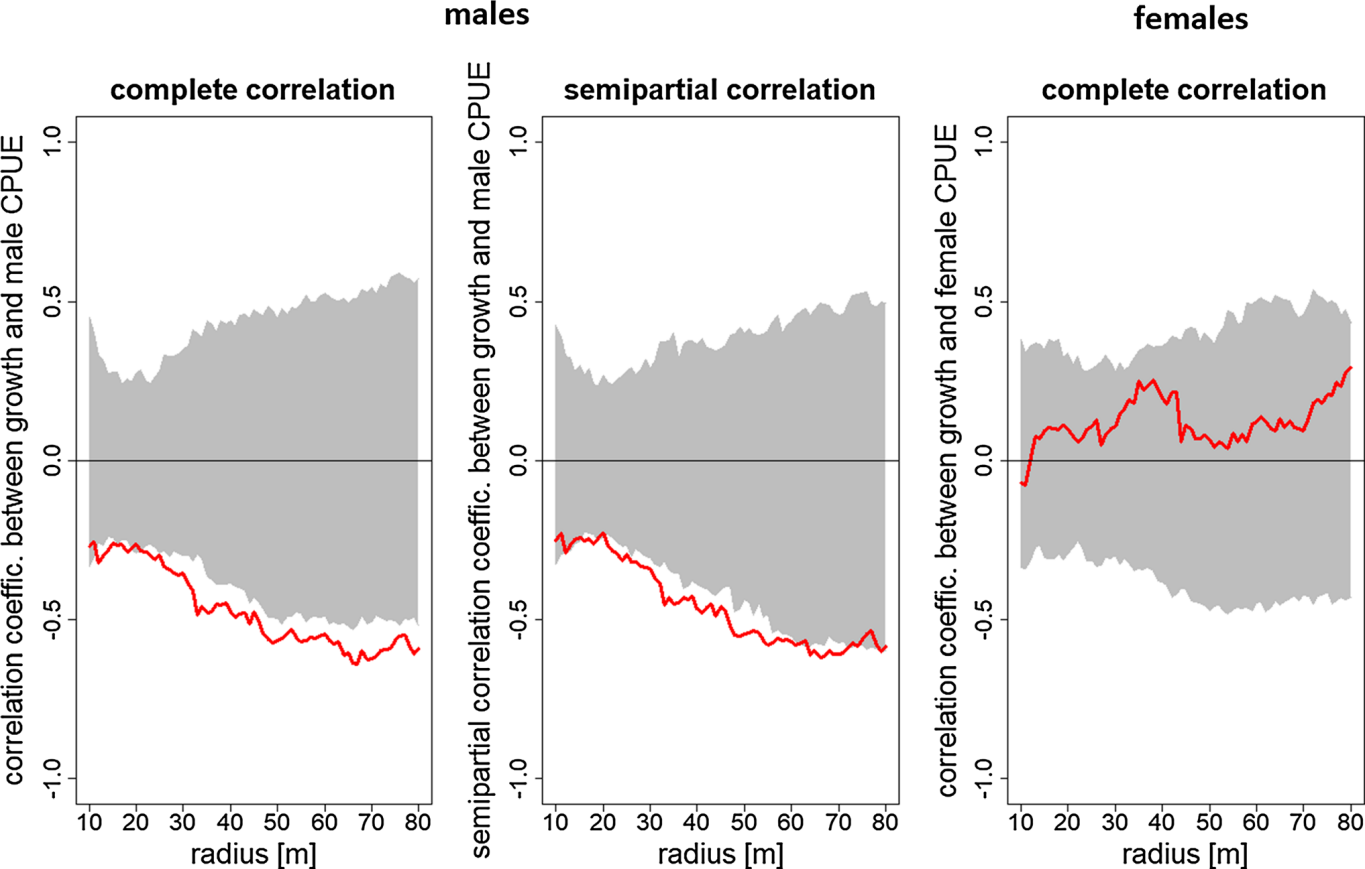
Correlation between CPUE and mean growth inside circles along different radii. Greyed area shows 2.5–97.5% percentile of Pearson correlation coefficient distribution calculated with permutation tests, red lines indicate sample values

Mean growth rates for males were negatively correlated with the local CPUE (Fig. 3). This pattern was evident and stronger than would be expected under randomisation for all scales examined. Semipartial correlations, controlling for different starting sizes, followed the same pattern. Density seemed to have disproportionate effects on the slower-growing males inside a sampling circle. Negative correlations between growth and CPUE were maintained when just using males in the lower quartile of growth. In contrast, correlations between growth and CPUE in faster-growing individuals were weakly negative and not more extreme than the expectations derived from randomisation (Suppl. Fig. S2). Overall, this implies competition where the males with slower growth rates are affected more by changes in density.

Growth rates for females did not seem to be associated with the local density as estimated with CPUE. Female correlations tended to be positive, but did not fall outside the confidence limits of permutations. As male and female CPUE had similar spatial patterns (Fig. 1b), it was unsurprising that results were similar using total CPUE (males + females), so only the results using CPUE calculated separately for males and females are presented here. The same results for males and females are shown using non-parametric (Spearman rank) tests, so any non-linear relationship between CPUE and density (e.g. Bell et al. 2001) does not alter these conclusions.

There were 533 recaptures of v-notched individuals over an approximately 2-month timeframe. Recapture rates were high: 36.6% of captured males and 43.1% of females bore a v-notch. While we were unable to distinguish the number of times a notched individual may have been recaptured, 13% of all individuals captured in a given day bore a v-notch, which indicated good survival. The catch of v-notched and non-v-notched individuals in 2014 was correlated (using the Dutilleul correction for spatial autocorrelation*, F* = 121.066, df = 1214.76, *p* < 0.01). In other words, probability for a v-notched recapture follows the expected rate based on total CPUE. This suggests density-independent survival rates for *Nephrops* on the fished grounds.

## Discussion

*Nephrops* demonstrates suitable characteristics for catch-and-wait fisheries enhancement. Retention of released individuals within the study area was high because individuals travelled relatively low distances from the release site in comparison to the size of the fished area (Table 1, Fig. 1) and their recovery was consistently proportional to fishing effort (RMSE plot—Suppl. Fig. S1). The distance travelled did not vary significantly by sex, starting size, growth rate (high or low), or whether individuals were at liberty for 1 or 2 years. In a related study, Haynes et al. (2016) described how individuals grew, on average, one size grade larger after catching and waiting for 1 year (until the following year’s fishing season).

Growth rates showed clear evidence for density dependence, but these effects were not homogeneous in the population, slower-growing males were the most affected by local density. Up to 2.7 mm carapace length compensation (expressed in reduced growth after ~ 1 year) was observed to occur in males for a CPUE difference of 0.7 individuals per pot (based on both sexes). Given that median growth per year is ~ 4.5 mm carapace length on average for males (Haynes et al. 2016), this represents a large compensation. Put another way, this represents a ~ 30% reduction compared to the maximal possible growth observed (95% percentile) in males. Although differences in *Nephrops* body sizes across different fishing grounds have been observed and correlated with density (Bailey 1986; Hillis and Tully 1993; Tuck et al. 1997; Johnson et al. 2013), the present study is the first to demonstrate the mechanism through which this happens, i.e. via density-dependent suppression of body size (growth). This satisfies an important criterion in resource management which is that, wherever possible, the mechanisms through which density dependence takes place should be specified (Rose et al. 2001).

Growth in *Nephrops* may be negatively affected by density due to direct aggression and interference between competing males. The peak of variance in growth within smaller sampling circles at scales between 10 and 15 m indicated that males with different growth rates were in close proximity to one another. In addition, the slower-growing individuals inside a sampling circle, as represented in the lower quartile plots (Suppl. Fig. S2), showed a significant negative correlation with density along various radii, but faster-growing individuals did not. Thus, competition was observed between ‘inferior’, i.e. slower-growing males, which were affected most by density and ‘superior’ faster-growing individuals. Sbragaglia et al. (2017) looked at aggressive interactions and the formation of dominance hierarchies in *Nephrops* and showed that these can be set up in less than 5 days. Dominant individuals also spent more time in their burrows (Sbragaglia et al. 2017), implying that they required less time to forage, which presumably enables them to divert more energy towards growth.

Females did not appear to be affected by density to the same extent as males. Growth slows down dramatically after sexual maturity in females (Haynes et al. 2016), so density-dependent effects on growth are more difficult to detect. Females appear to place energy into reproduction rather than growth once a threshold size has been reached, whereas males may continue to benefit from high growth rates post-maturity if this ensures optimal access to burrows, food, and mates. There was unexpected heterogeneity in growth rates when examining sampling circles below 20 m radius. This may indicate that some competitive interactions exist in females, but these are not intensified by density, perhaps due instead to habitat quality—density trade-offs under an ideal free distribution (Katoh et al., 2013 provides further reading on behavioural interactions).

The existence of density compensation informs management on trawling grounds, for example, our results indicate that fishing pressure on high density grounds may alleviate density suppression of body sizes (individual biomass). Indeed, density dependence is a critical concept inherent in the ‘surplus’ of surplus yield models used to predict appropriate levels of fishing mortality. However, with notable exceptions (Ricker 1954; Beverton and Holt 1957), evidence of density-dependent effects in marine populations remains highly controversial. One of the problems articulated by Rose et al. (2001) is that few studies have conducted experiments which measure the mechanism and magnitude of density-dependent processes over a time series. By contrast, in terrestrial systems, density dependence has been very tightly linked to resource management and for certain monocultures, e.g. forestry plantations, has been linked to a carrying capacity as described by the -3/2 self-thinning ‘law’ (Yoda et al. 1963; but see also LaBarbera 1989).

### Development of catch-and-wait fisheries

Alongside suitable species and fishing techniques, catch-and-wait fisheries probably need bioeconomic evaluations to determine where they can be developed. Optimal return size will be price- and growth rate-dependent. At some point it is inefficient to throw back large individuals since these have less scope for growth (Haynes et al. 2016) and these should be landed instead. Where more than one fisher is involved in a local area, catch-and-wait needs management via fisheries cooperatives, to enable fair access for those engaged in the enterprise. Creel-caught *Nephrops* are exempt from the Landings Obligation (European Commission 2013) due to good survivability (Méhault et al. 2016). The operation of catch-and-wait fisheries in inshore areas is not likely to substantially increase food production, rather this may supplement and enhance inshore stocks and widen the options available to small-scale fishers in peripheral coastal communities with few employment opportunities. Size-selective harvesting and fisheries-induced evolution is of increasing concern (e.g. Enberg et al. 2012); however, the potential for such evolutionary effects to arise due to catch-and-wait practices is probably negligible in *Nephrops* because of the localised scale of these practices relative to the large dispersal capability in this species (Stamatis et al. 2004; O’Sullivan et al. 2015).

*Nephrops* capture fisheries have high economic importance in Europe but have been subject to declines in some functional units (e.g. Aran Grounds, Porcupine Bank and Southern Biscay; Ungfors et al. 2013). Similarly, American lobster *Homarus americanus* has become the most valuable fishery in North America but large stock fluctuations have been seen in some areas. Such variability has been linked to global warming (Le Bris et al. 2018), altered food web structure arising from overfishing finfish stocks (Steneck and Wahle 2013), and disease (Wahle et al. 2009). Interestingly, a positive feedback between pot fishing intensity and American lobster abundance suggests that bait used in the fishery may itself have contributed to increased landings (Saila et al. 2002). Juvenile lobsters can enter and exit pots, with bait consumed while inside the pot calculated to provide a subsidy equivalent to between one-quarter and one-third of lobster landings in the Gulf of Maine (Saila et al. 2002). Subsidy has interesting theoretical and practical applications: fishing activity and the use of bait may increase the local carrying capacity. Considering a species with good site fidelity like *Nephrops*, the subsidy from bait illustrates how wild stocks could be ‘farmed’ on local mud patches. Indeed, moribund fish or crabs or molluscs by-caught in pots (pers. obs.) may potentially augment fishing bait subsidies.

## Conclusion

The catch-and-wait fishery has supplied detailed, spatially explicit information on movement and growth of *Nephrops* in the field, demonstrating the circumstances where density-dependent effects on growth occurred. The large density compensation seen in male growth indicates resilience to fishing pressure (even though males are more available to year-round fishing than are females), and provides a mechanism for the existence of different sizes of lobsters on fishing grounds with different densities. These processes may also be important for conservation measures. We provide empirical support for modelling studies (e.g. Gårdmark et al. 2006) which show that protections inside marine reserves or no-take zones that increase densities can lead to reduced body growth, as a consequence of density dependence. Density dependence should therefore be taken into account for conservation planning of marine reserve and no-take-zones.

## Electronic supplementary material

Below is the link to the electronic supplementary material.

Supplementary material 1 (PDF 416 kb)
