# Supplementary material for: Density-dependent growth in ‘catch-and-wait’ fisheries has implications for fisheries management and Marine Protected Areas
Source: Ambio. 2019 Mar 9;49(1):107–17. doi: 10.1007/s13280-019-01158-1 (PMC6889112; doi:10.1007/s13280-019-01158-1)
Supplement: Supplementary file 1 — Supplementary material 1 (PDF 416 kb) [file 13280_2019_1158_MOESM1_ESM.pdf]

Ambio

Electronic Supplementary Material

This supplementary material has not been peer-reviewed.

Title: Density dependent growth in ‘catch-and-wait’ fisheries has implications for fisheries management and Marine Protected Areas

Authors: Julian Merder, Patricia Browne, Jan A. Freund, Liam Fullbrook, Conor Graham, Mark P. Johnson, Alina Wieczorek, Anne Marie Power

Materials and methods (additional Information):

Kernel density estimation:

Kernel density estimates (Fig. 1 B) were calculated with a Gaussian kernel with a bandwidth of 70 m, which also matched the optimal bandwidth suggestions by Stoyan and Stoyan (1995) and with edge correction (Jones 1993). We used a grid of ~15,000 cells inside a polygonal window isolating the study area (Fig. 1 A) to avoid steep cuts over the area.

We computed separate estimates for pot density, from pot position data and for total catch density, from the recapture data (tagged and untagged). Population density was estimated from CPUE obtained at each grid point as the ratio of total catch density and pot density.

Bandwidth selection of the Nadaraya-Watson smoother (Nadaraya 1964; Nadaraya 1989; Watson 1964) was based on default cross-validation methods, cells were chosen identical with those of the CPUE grid of total catch (Fig. 1 B).

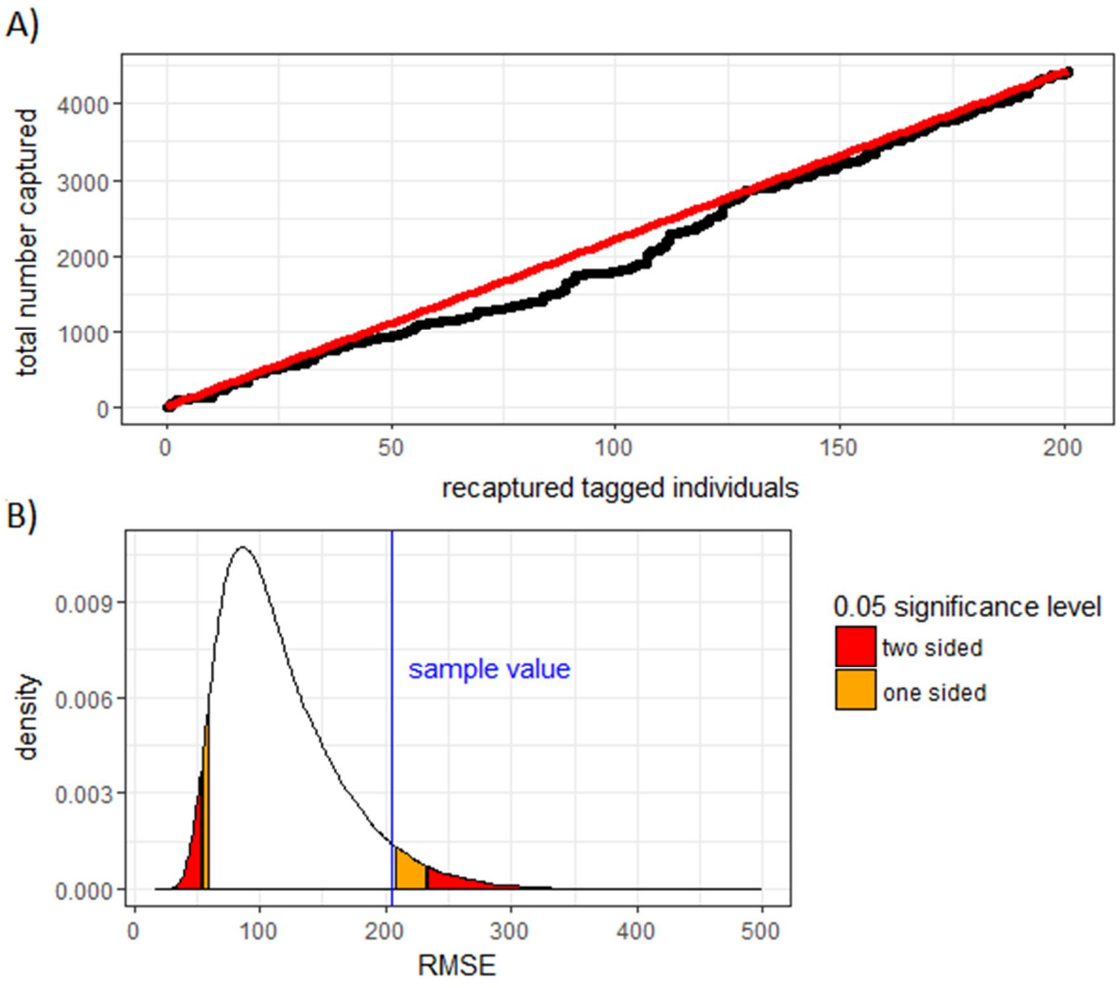

Supplementary Figure S1: A) Total number of captures versus total number of recaptured tagged *Nephrops norvegicus* individuals shows a linear relationship. Root Mean Square Error (RMSE) from the line of constant recapture (red, slope calculated from ratio of total catch and total tagged recaptures) does not differ significantly from RMSE values obtained by permutation tests (see B & main text). B) Density plot showing the distribution of RMSE values obtained (in Fig S1 A) by permutation tests.

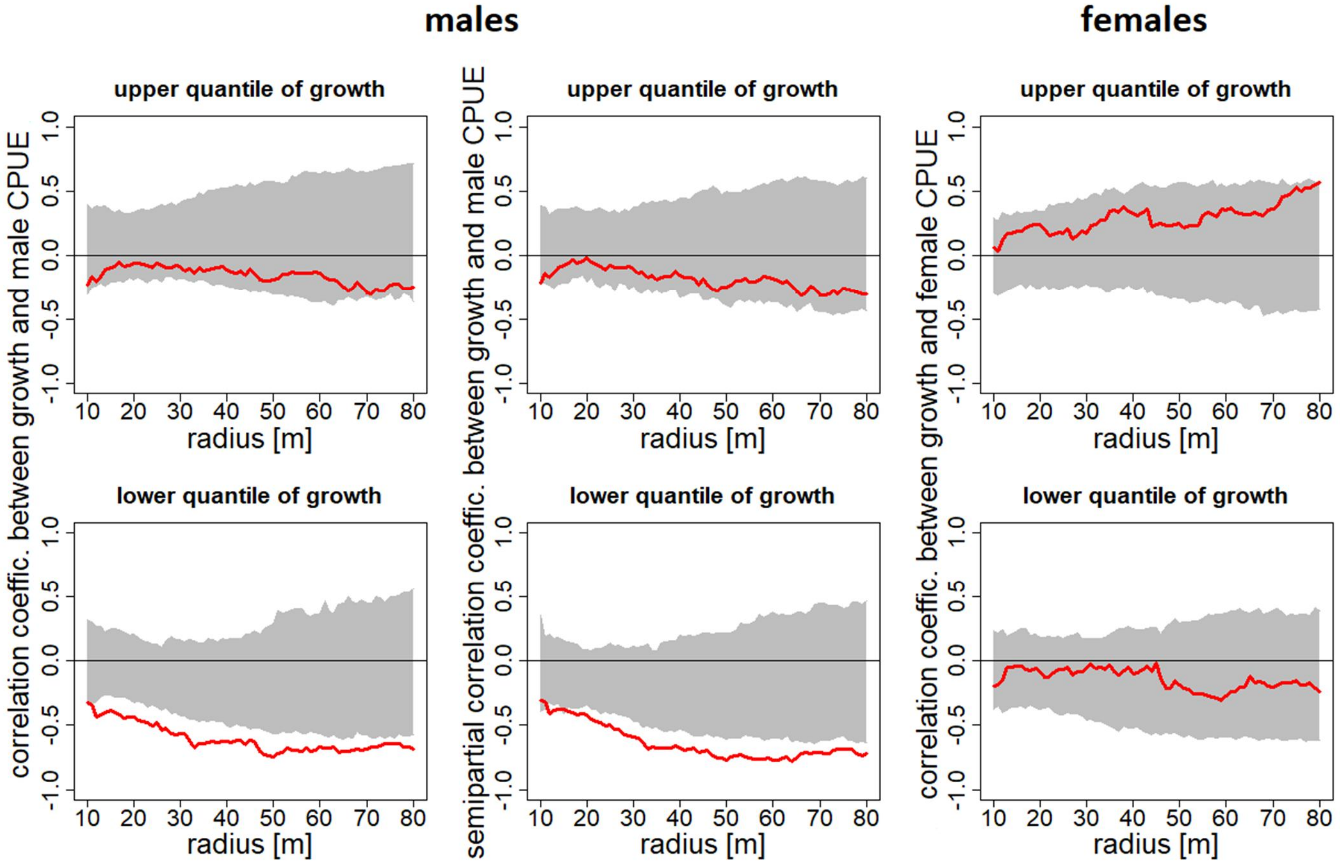

Supplementary Figure S2: Correlation between CPUE and upper/lower quartiles of *Nephrops norvegicus* growth inside circles along different radii for males and females. Greyed area shows 2.5-97.5% percentile of Pearson correlation coefficient distribution calculated with permutation tests, red dots indicate sample values.

References cited:

Jones, M.C. 1993. Simple boundary corrections for kernel density estimation. *Statistics and Computing* 3: 135—146.

Nadaraya, E.A. 1964. On estimating regression. *Theory of Probability and its Applications* 9: 141–142.

Nadaraya, E.A. 1989. Nonparametric estimation of probability densities and regression curves. *Mathematics and its Applications (Soviet Series) 20*. Dordrecht: Springer.

Stoyan, D., Stoyan, H. 1995. *Fractals, random shapes and point fields: methods of geometrical statistics*. Chichester: John Wiley and Sons.

Watson, G.S. 1964. Smooth regression analysis. *Sankhya: The Indian Journal of Statistics Series A* 26: 359–372.
